# The 17th Rocky Mountain Virology Association Meeting

**DOI:** 10.3390/v9110333

**Published:** 2017-11-08

**Authors:** Joel Rovnak, Rushika Perera, Matthew W. Hopken, Jenna Read, Derrick M. Waller, Randall J. Cohrs

**Affiliations:** 1 Department of Microbiology, Immunology and Pathology, Colorado State University, Fort Collins, CO 80523, USA; Joel.Rovnak@colostate.edu (J.R.); mhopken@rams.colostate.edu (M.W.H.); 2 Arthropod-borne and Infectious Diseases Laboratory, Department of Microbiology, Immunology and Pathology, Colorado State University, Fort Collins, CO 80523, USA; rushika.perera@colostate.edu (R.P.); jlread@rams.colostate.edu (J.R.); 3 USDA-APHIS National Wildlife Research Center, Fort Collins, CO 80521, USA; 4 Department of Pharmaceutical Sciences, Regis University School of Pharmacy, Denver, CO 80221, USA; dwaller@regis.edu; 5 Departments of Neurology and Immunology/Microbiology, University of Colorado School of Medicine, Aurora, CO 80045, USA

**Keywords:** prions, viruses, Dengue, Sinbis, CWD, Zika, metabolism, herpesvirus, primate, transmission

## Abstract

Since 2000, scientists and students from the greater Rocky Mountain region, along with invited speakers, both national and international, have gathered at the Mountain Campus of Colorado State University to discuss their area of study, present recent findings, establish or strengthen collaborations, and mentor the next generation of virologists and prionologists through formal presentations and informal discussions concerning science, grantsmanship and network development. This year, approximately 100 people attended the 17th annual Rocky Mountain Virology Association meeting, that began with a keynote presentation, and featured 29 oral and 35 poster presentations covering RNA and DNA viruses, prions, virus-host interactions and guides to successful mentorship. Since the keynote address focused on the structure and function of Zika and related flaviviruses, a special session was held to discuss RNA control. The secluded meeting at the foot of the Colorado Rocky Mountains gave ample time for in-depth discussions amid the peak of fall colors in the aspen groves while the random bear provided excitement. On behalf of the Rocky Mountain Virology Association, this report summarizes the >50 reports.

## 1. Introduction

**Figure 1:**
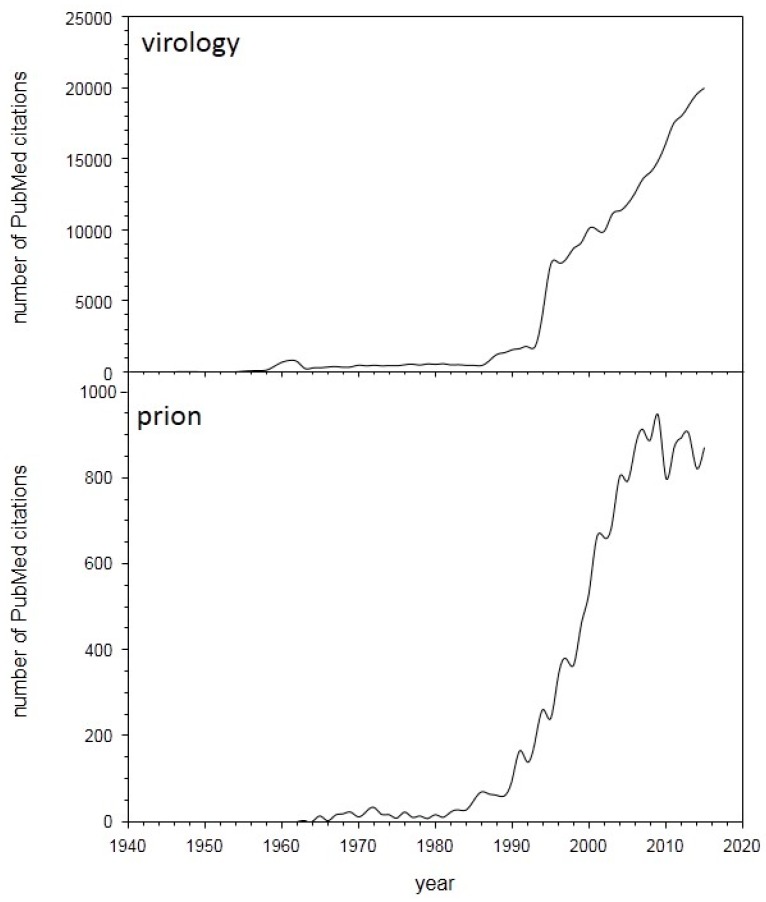
PubMed citations by year using virology (**top** panel) and prion (**bottom** panel).

**Figure 2:**
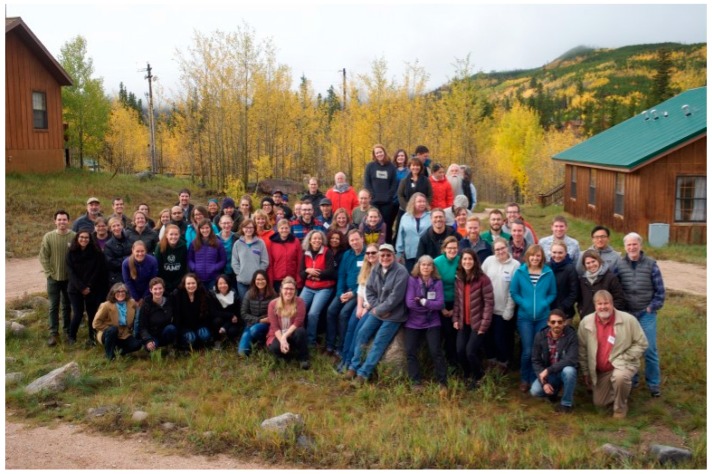
Attendees at the 2017 meeting of the Rocky Mountain Virology Association in the Mountain Campus of Colorado State University.

As can be seen by a graph depicting the number of PubMed citations per year, advances in virology and prion studies are accelerating at a pace that makes it difficult for any individual to remain informed in areas outside one’s specialty (Figure 1). In addition, scientific meetings are continuing to focus on specific areas to maximize dissemination of information to select groups while general meetings that cover multiple fields are typically too large to permit prolonged informal discussion, especially with students and early-stage investigators. With these facts in mind, the Rocky Mountain Virology Association (RMVA) was formed to provide a venue that permitted formal presentations of current research in multiple areas of virology and prion biology in a venue that is sufficiently removed from major cities to ensure extended informal discussions and opportunities to establish/strengthen collaborations. Professional child daycare was provided to help enable attendance by individuals with young children, which provided an educational opportunity through the children’s participation in a virus and prion themed performance during the formal poster session. Taken together, the 17th annual RMVA meeting (Figure 2) upheld the tradition of presenting novel findings, summarized below, that spanned the fields of bio-informatics, host-pathogen interactions, immunology, therapeutics, replication of DNA and RNA viruses along with prion detection and disease propagation.

## 2. Summary of Scientific Sessions

The tenor of the 2017 meeting was set with the keynote presentation provided by Richard J. Kuhn (Department of Biological Sciences and Director, Purdue Institute of Inflammation, Immunology and Infectious Disease, Purdue University) who described his team’s high resolution anatomical study of flaviviruses. Zika virus emerged in 2015–2016 in the Americas, with rapid spread and substantial impact. His team solved the structure of this flavivirus virion to near atomic resolution, and this revealed the similarity with previous flavivirus structures such as dengue virus and West Nile virus. Despite strong overall structural similarity, regions of the envelope (E) glycoprotein revealed differences that suggested sequences that might be involved in the unique cell and tissue tropism that Zika has displayed. Together with dengue virus, his team continued to probe the structure and composition of these viruses and their assembly intermediates using the tools of cryo-electron microscopy and mass spectrometry. In particular, they probed the process of virus particle maturation examining the immature virus particle and the conversion of prM by cellular furin to the mature M form of the protein. This process in dengue virus is very inefficient and they determined the stoichiometry of prM/M proteins in the Zika virion, and evaluated the implications of the maturation state on virus biology. Antibody binding to the particle has also been examined and has revealed insights into the process of virus maturation as well as entry of the virion into target host cells. The ability to compare virus particles from multiple members of the flavivirus genus in terms of structure and function serves as a powerful strategy to discern common processes and distinct features that contribute to virus biology.

### 2.1. RNA Viruses

**Reyes A. Murrieta** (Arthropod-Borne and Infectious Diseases Laboratory, Department of Microbiology, Immunology and Pathology, CVMBS, Colorado State University, Fort Collins, CO, USA) described his work on the impact of environmental conditions on defining the population diversity of Zika virus in *Aedes* mosquitoes. The emergence of arthropod-borne viruses (arboviruses) has become an ever-growing public concern, highlighted by the recent emergence of Chikungunya virus and Zika virus (ZIKV) in the Americas. The conditions that lead to the emergence of new virus genotypes are poorly understood. In mosquitoes, temperature profoundly impacts virus transmission via replication rate and the likelihood that virus will disseminate from the midgut and ultimately be transmitted. Accordingly, they sought to define the impact of temperature on transmission dynamics of ZIKV and determine whether temperature affects virus population structure during extrinsic incubation (EI) in the mosquito. In particular, they exposed *Aedes aegypti* and *Aedes albopictus* mosquito vectors to a bloodmeal containing a Puerto-Rican strain of Zika virus (ZIKV, *Flaviviridae*, *Flavivirus*) and held the mosquitoes for 7 and 14 days at 25 °C, 28 °C and 35 °C. They also exposed the infected mosquitoes to fluctuating temperatures that varied from 25 to 35 °C diurnally. They found that transmission of ZIKV in both *Aedes* mosquitoes was most efficient at 28 °C (*p* < 0.05). However, viral load in the midgut and hemolymph was highest at 35 °C (*p* < 0.05). Using RNA deep sequencing (RNAseq) to characterize virus genetic diversity, they described that ZIKV populations in the midgut, hemolymph, and saliva were shaped by the EI temperature. Collectively, their data indicated that temperature has multiple impacts on ZIKV biology.

**Claudia Rückert** (Arthropod-borne and Infectious Diseases Laboratory, Department of Microbiology, Immunology and Pathology, College of Veterinary Medicine and Biomedical Sciences, Colorado State University, Fort Collins, CO, USA) described her work on the evolution of chikungunya, dengue and Zika viruses in single, dual and triple infected *Aedes aegypti* mosquitoes. Recent evidence suggests that coinfection with chikungunya (CHIKV), dengue (DENV) and Zika (ZIKV) viruses may be relatively common in areas where all three viruses are co-circulating, including large parts of the Americas. Here they showed that coinfection of *Aedes aegypti* (Poza Rica) mosquitoes with all combinations of the three viruses is possible, and co-transmission may occur with little impact on vector competence. Only subtle differences in vector competence were observed and viral load was comparable in saliva. However, slightly reduced viral load was observed in coinfected mosquito bodies and legs. They were thus interested in characterizing interactions between the three viruses in the mosquito. In order to understand how coinfection impacts virus evolution, *Aedes aegypti* (Poza Rica, Mexico) were exposed to blood meals containing more than one *Aedes*-borne arbovirus. Specifically, mosquitoes were orally exposed to American strains of CHIKV (99659), DENV-2 (Merida), or ZIKV (PRVABC59) in single infections, as well as combinations of the three viruses as double and triple infections. Mosquitoes were held for 12 days extrinsic incubation, at which time mosquito saliva, salivary glands, legs and midguts were collected. Samples were processed for RNAseq on the Illumina HiSeq4000 platform. They analyzed changes to viral consensus sequences and virus population structure in single versus coinfected mosquito tissues. Overall, their data suggested that infection with more than one arbovirus simultaneously may affect virus evolution in *Aedes aegypti* mosquitoes.

**Rebekah C. Gullberg** (Arthropod-borne and Infectious Diseases Laboratory, Department of Microbiology, Immunology and Pathology, Colorado State University, Fort Collins, CO, USA) initiated a conversation about host lipids and their ability to alter the infectivity of dengue virus, serotype 2 particles. As obligate intracellular parasites with limited genetic capacity, viruses must find creative ways to use cellular resources for their replication. Rearranging the structure of cellular membranes is a key requirement for positive-strand RNA viruses. There are multiple ways to rearrange a phospholipid membrane, such as controlling its source (e.g., particular organelle) or by altering cellular lipid biosynthesis to concentrate or deplete a given lipid species. Dengue viruses are the most aggressive arthropod-borne positive-strand RNA viruses in the world. It is well understood that dengue virus type 2 (DENV2) alters fatty acid metabolism during infection in the mosquito and human hosts. Fatty acids are a key component of endoplasmic reticulum (ER) membranes utilized for the viral life cycle. The Perera lab showed that DENV specifically alters the content of unsaturated fatty acids (UFA) in cellular membranes. In order to accomplish this, DENV2 upregulates and recruits Stearoyl CoA-desaturase (SCD), the rate-limiting enzyme in UFA biosynthesis, early during infection to sites of viral replication. Knockdown and inhibition of SCD resulted in reduced viral titers. Furthermore, they found that in the absence of SCD activity, DENV2 was less infectious in the next round of infection which led to the hypothesis that the virus titer reduction was due to a change in the content of the lipid envelope, which produced immature particles. Despite the lack of virus-derived lipid biosynthetic machinery, DENV2 was able to drastically alter cellular lipid content by manipulating SCD expression and thus produce mature virions. Hence they speculate that UFA biosynthesis pathway is a promising target for therapeutic intervention.

**Nunya Chotiwan** (Arthropod-borne and Infectious Diseases Laboratory, Department. of Microbiology, Immunology and Pathology, Colorado State University, Fort Collins, CO, USA) continued the conversation on host lipids during dengue virus infection and presented data on how the ceramide and dihydroceramide ratio could impair dengue virus serotype 2 infection of *Aedes aegypti*-derived cells. Sphingolipids (SPs) are bioactive molecules that play roles in the structural composition of cellular membranes and cell-signaling pathways. SPs have also been highlighted in many studies as a lipid metabolic pathway altered by flavivirus infection. In the metabolomics study of midguts from *Aedes aegypti* mosquitoes infected with dengue virus (DENV) type 2, The Chotiwan lab identified and reconstructed a significant proportion of the SP pathway. This was the first functional annotation and validation of the pathway. They observed an accumulation of precursors and downstream derivatives of ceramide (CER), a hub of the SP pathway, during DENV infection. The balance between CER and its precursor, dihydroceramide (DHCER), plays critical roles in membrane architecture, fluidity and function. Therefore, they investigated whether modulation of CER and DHCER levels and the CER/DHCER ratio would have an impact on DENV infection in *Aedes aegypti*-derived cells using loss-of-function studies and validated the changes in metabolites by tandem mass-spectrometry. Cells were treated with 4-Hydroxyphenyl Retinamide (4HPR) or long double-stranded RNA (dsRNA) to inhibit activity of or knockdown the expression of sphingolipid Δ-4 desaturase (DEGS), the enzyme that converts DHCER to CER. Inhibition of DESG by 4HPR showed significant reduction of virus titer and genome replication but did not alter CER/DHCER ratios. However, DESG expression knockdown by dsRNA reduced DENV infection and altered CER/DHCER ratios. These results indicated that functionality of DEGS is important for DENV infection and the balance of CER and DHCER is a potential target for disrupting DENV transmission in *Aedes aegypti* mosquitoes.

**Kirsten Krieger** (Arthropod-borne and Infectious Diseases Laboratory, Department. of Microbiology, Immunology and Pathology, Colorado State University, Fort Collins, CO, USA) kept the “lipid-centric” conversation going and described how phosphatidylcholine-enriched membrane environments influenced the replication of dengue viruses. Dengue Viruses (DENV) are positive strand RNA viruses that account for almost 400 million infections annually making them the most aggressive arthropod-borne human pathogens in the world. Positive strand RNA viruses share the common characteristic of reorganizing host cellular phospholipid membranes to assemble their viral replication complexes (VRCs). Unfortunately, how these viruses regulate membrane reorganization is unknown. During DENV replication, dramatic changes occur in lipid biosynthesis, that have a significant impact on membrane architecture and cellular signaling pathways. Phosphatidylcholine (PC) is a major phospholipid component of many lipoproteins, and makes up about 50% of total phospholipid content in cellular membranes. PC is synthesized in the endoplasmic reticulum via two distinct pathways: the Kennedy pathway or the PEMT pathway. The Kennedy pathway, abundant in most tissues, includes a crucial step in which phosphocholine is converted to CDP-choline by the enzyme, CTP: phosphocholine cytidylyltransferase (CCT*alpha*). CCT*alpha*, encoded by the *PCYT1A* gene, is the rate-limiting enzyme for PC synthesis. Studies on Brome Mosaic Virus (BMV), another positive strand RNA virus, have shown that BMV stimulates PC synthesis at viral replication sites. It has also been documented that PC accumulates in DENV infected mosquitoes and mosquito cells as well as during Hepatitis C and poliovirus infection. Preliminary results by the Krieger lab supported their hypothesis that dengue virus uses the CCT*alpha* enzyme to increase and regulate phosphatidylcholine synthesis during viral replication in order to change the lipid composition of host cellular membranes to benefit the viral life cycle.

**Jenna Read** (Arthropod-borne and Infectious Diseases Laboratory, Department. of Microbiology, Immunology and Pathology, Colorado State University, Fort Collins, CO, USA) provided an update concerning fatty acid chain elongation in human cells impacting on the replication of dengue virus, serotype 2. Dengue viruses (DENV) are arthropod borne viruses with widespread impact consisting of 390 million new cases every year. Due to a geographical shift in arbovirus spread, viruses such as West Nile, dengue, and now Zika are becoming increasing threats to the United States. In order to develop antiviral or therapeutic agents, it is important to understand how the virus interacts with the host to initiate infection, replication and transmission. As obligate parasites, viruses require metabolic resources from host cells for productive infection. Many viruses including DENV impact the flux from glucose to acetyl-CoA to feed fatty acid metabolism in infected cells. For this purpose, they hijack several enzymes responsible for the biosynthesis or degradation of fatty acids. Saturated very-long chain fatty acids (VLCF) are one group of fatty acids that are hijacked by many viruses. Mammals have seven elongase enzymes to synthesize saturated VLCFs. Preliminary data on dengue virus has indicated that Elongation of Long-Chain Fatty Acid Family Member 6 (ELOVL6) is important for virus replication. ELOVL6 has been identified as a rate limiting enzyme that catalyzes the elongation of saturated and unsaturated long-chain fatty acids. These fatty acids can be used as precursors for more complex cellular membrane lipid production. The Read lab is validating the role of this enzyme during dengue virus replication under the hypothesis that dengue virus requires ELOVL6 to increase membrane fluidity and support the formation of membrane-bound virus replication complexes, and that blocking the activity of ELOVL6 may be a potential avenue for antiviral intervention.

**Elena Lian** (Arthropod-borne and Infectious Diseases Laboratory, Department. of Microbiology, Immunology and Pathology, Colorado State University, Fort Collins, CO, USA) presented preliminary data on using fatty acid desaturase 2 (FADS2) enzyme activity as a control point to limit dengue virus serotype 2 replication. Dengue is an arthropod-borne viral disease with an estimated 400 million infections annually across the globe. Dengue fever is caused by dengue viruses (DENV) and despite the prevalence of this disease, there are currently no US Food and Drug Administration (FDA)-approved vaccines or antivirals. DENV has been shown to manipulate the host’s lipid biosynthesis pathways, specifically that of unsaturated fatty acids, for the assembly and function of viral replication complexes. Lipids are also used as cell signaling molecules for purposes such as antiviral defense mechanisms. By controlling lipid biosynthesis, DENV can better evade the host’s immune response. One of the major lipid pathways altered by DENV is alpha-linolenic acid and linoleic acid metabolism. Arachidonic acid is one of the products from this pathway, and is an inflammatory molecule involved in cell signaling. Since DENV incites an inflammatory response, this pathway is an ideal drug target. Fatty acid desaturase (FADS2) is the rate-limiting enzyme used to catalyze the pathway; therefore, it is a potential target to reduce DENV replication. Preliminary evidence indicated a reduction in DENV replication in human cells through the inhibition of FADS2. To test the hypothesis that inhibition of FADS2 will impede DENV replication by reducing the presence of arachidonic acid in the cell from linoleic acid metabolism, the Lian lab plans to further investigate how inhibition of FADS2, with an inhibitor and with siRNA, will affect DENV, as well as cellular viability and function.

**Stephanie Mills** (Arthropod-borne and Infectious Diseases Laboratory, Department. of Microbiology, Immunology and Pathology and Department of Biological Sciences, Colorado State University, Fort Collins, CO, USA) discussed the potential of using Acyl-CoA thioesterases as restriction factors of dengue viruses. Although dengue viruses (DENV) infect over 400 million people per year, only a suboptimal vaccine exists, and other therapeutic options are unavailable. Previous studies indicate that DENV utilizes lipids to replicate in host cells and preliminary loss of function analyses of fatty acid metabolizing enzymes identified Acyl-CoA thioesterase-1 (ACOT-1) to significantly increase DENV particle release. ACOT-1, a 42 kDa enzyme which hydrolyzes C-12 to C-20 length Acyl-CoA fatty acids, has been shown to reduce sepsis and prevent diabetes. The Mills’ lab analyzed the role ACOT-1 in the life cycle of DENV. They harvested Huh-7 cells infected with DENV at 3 h intervals for 48 h. Uninfected cells and UV-inactivated DENV-exposed cells were included as controls. They analyzed ACOT-1 protein levels compared to viral protein NS3 in cell lysates on western blots and also analyzed the impact of siRNA mediated knockdown of ACOT-1 on DENV RNA replication by electroporating a luciferase reporter-tagged viral replicon following siRNA treatment. Validation of ACOT-1 messenger RNA (mRNA) levels in knockdown and control cells were done by quantitative real-time polymerase chain reaction (qRT-PCR). Localization of ACOT-1 protein upon infection was determined using immunofluorescence microscopy. Although ACOT-1 mRNA levels were inconclusive, ACOT-1 protein levels increased and then spiked between 9 and 12 h post infection, before decreasing to base line levels 21 h post infection. DENV RNA levels increased five-fold above controls upon siRNA mediated knockdown of ACOT-1. However, unlike other fatty acid metabolizing enzymes in DENV infected cells, ACOT-1 did not co-localize with NS3 upon infection. Their results indicated there was an increase in DENV particle release and genome replication upon loss of ACOT-1 function. Therefore, ACOT-1 may be the first fatty acid enzyme that plays a protective role in host cells against DENV infection.

**Caroline Montgomery** (Arthropod-borne and Infectious Diseases Laboratory, Department of Microbiology, Immunology and Pathology and Department of Biological Sciences, Colorado State University, Fort Collins, CO, USA) described her analysis of exosomes released from Zika virus infected cells as a paracrine signaling mechanism to prime neighboring cells for enhanced infection. Cell-to-cell communication has the capacity to enhance viral infection by releasing signaling molecules or vesicles from infected cells that prime recipient cells. One way these communication pathways can function is by giving an advantage to the virus as it replicates by priming the neighboring cells and making them more susceptible to infection. The signaling molecules can circulate in the host and give way to prolonged consequences of infection. These molecules are referred to as the secretome of cells and may contain lipids, nucleic acids and proteins and can be transported by extracellular vesicles. For example, fatty acid synthase (FAS), which is an important enzyme in lipid metabolic pathways, can be transported by extracellular vesicles to increase the metabolism of the recipient cells. FAS is required in the viral lifecycle, and through transport by these vesicles, it can metabolically activate the recipient cells and prime them for infection. Through this observation, it is suggested that infected cells can participate in paracrine signaling, thus enhancing viral replication in neighboring cells. Under the hypothesis that vesicles released by ZIKV infection will enhance the metabolism of neighboring cells, thus increasing the ability of ZIKV to replicate in these cells, the Montgomery lab presented preliminary data concerning secretome of ZIKV infected cells to determine whether they are able to participate in paracrine metabolic activation.

**Alison Gilchrist** (Department of Molecular, Cellular, Developmental Biology and the Biofrontiers Institute, University of Colorado, Boulder, CO, USA) shifted the conversation to host protein targets predicted to be cleaved by the dengue virus protease. Dengue virus translates its genome into a single polyprotein and uses a self-encoded protease to cleave the polyprotein into individual proteins. The target site specificity for this protease is loose, so The Gilchrist lab anticipated that it also cleaved some host proteins in a manner that may be important for the viral life cycle. Using a machine learning approach, they have created a predictor to search for Dengue protease cleavage sites within the human proteome. They have experimentally validated that several human proteins in this candidate list were cleaved by the Dengue protease, and were important for replication. They provided insight now that each of these host-virus interactions may be acting as species-barriers in the movement of viruses between primate species, and which of these genes may be evolving rapidly in primate populations; information that was tested experimentally with different orthologs in Dengue infection assays. This project demonstrated how analysis tools from the field of molecular evolution could help facilitate novel characterization of host-virus biology.

**Abhilash Chiramel** (National Institute of Health/Rocky Mountain Laboratories, Hamilton, MT, USA) introduced the audience to the concept that human TRIM5 protein could function as a host-derived restriction factor for flaviviruses. Tripartite motif (TRIM) proteins are interferon-inducible genes (ISGs) and function as virus- and host-specific restriction factors that can interact with viral proteins and inhibit viral replication. Flaviviruses, include globally significant human pathogens such as tick-borne encephalitis virus (TBEV), West Nile virus (WNV) and dengue virus. TBEV replication is inhibited by TRIM30D, a rodent-specific ISG that functions by binding and targeting the viral RNA polymerase (NS5) for lysosomal degradation. TRIM5 a retrovirus-specific restriction factor is the closest human paralog of TRIM30D. The Chiramel lab showed that both human (h) and rhesus (rh) TRIM5 restricted the replication of viruses belonging to the TBEV serogroup, but not WNV. Ectopic expression rhTRIM5 on TBEV significantly reduced the production of infectious virus by more than 99%. hTRIM5 overexpression imposed a 90% reduction of virus titer, whereas knock-down of hTRIM5 partially rescued the antiviral effects of interferon treatment. TRIM5 reduced production of both viral RNA and protein, indicating that TRIM5 targeted an early step in virus replication. However, unlike TRIM30D, TRIM5 did not interact with NS5 and instead interacts with and degrades NS3, an RNA helicase critical to virus replication, by a mechanism involving the proteasome. Moreover, TRIM5 colocalized with NS3 and dsRNA suggesting that TRIM5 disrupts the viral replication complex. This work demonstrated that TRIM5 could target two very different viral proteins, the human immunodeficiency virus (HIV) capsid, and the TBEV helicase. Understanding the genetic trade-offs in TRIM5 that enable restriction of one virus versus another will illuminate how the evolution of host resistance is shaped by multiple pathogens.

**Cody J. Warren** (BioFrontiers Institute, Department of Molecular, Cellular, and Developmental Biology, University of Colorado Boulder, CO, USA) discussed his work on the cluster of differentiation 4 (CD4) receptor and how it restricts cellular entry of human immunodeficiency virus (HIV)-1 in nonhuman primates. Acute-stage isolates of HIV-1 require two receptors to enter cells, CD4 and C-C chemokine receptor type 5 (CCR5). The surface glycoprotein of HIV-1, Envelope (Env), mediates interaction with these two receptors facilitating virus entry into cells. The Warren lab showed that HIV-1 is highly adapted to use human CD4, and that nonhuman primate forms of CD4 restrict HIV-1 entry. Interestingly, HIV-1 poorly enters cells bearing even chimpanzee or gorilla CD4, despite the close relationship of these species to humans. They identified the reason why this important host restriction in nonhuman primates has been underappreciated to date: virions bearing Envs commonly used in SHIVs (SIV/HIV chimeric viruses), which are isolated from chronic-stages of infection and often are laboratory-adapted, efficiently infect cells expressing both human and nonhuman primate CD4 receptors. The finding that HIV-1 is highly adapted to humans in its use of CD4 has the potential to impact how HIV-1 infection and transmission is modeled in nonhuman primates.

**Joseph A. Westrich** (Department of Immunology and Microbiology, University of Colorado School of Medicine, Aurora, CO, USA) provided evidence that the high-risk human papillomavirus E7 stabilizes the APOBEC3A protein by inhibiting Cullin-dependent protein degradation. Recent studies have revealed abundant APOBEC3 signature mutations in human papillomavirus (HPV)-positive cervical and head and neck cancers, suggesting a role in HPV-positive cancer progression. The Westrich lab and others have shown that A3A, a member of the APOBEC3 family, is upregulated in HPV-positive keratinocytes and cancer patient tissues. They sought to determine if the HPV oncoprotein E7 had a post-translational effect on the A3A protein, thus they determined A3A protein levels in human keratinocytes expressing various HPV E7s. They found that high-risk HPV (HPV16 and 18) E7s, but not low-risk HPV (HPV6 and 11) E7s, inhibit A3A proteasomal degradation, effectively stabilizing the A3A protein. This effect was mediated by the motifs in the domain of HPV16 E7 involved with interaction with Cullin 2 (CUL2), a core component of the E3 ubiquitin ligase complex. Knockdown of CUL2, or inhibition of Cullin activation, resulted in protecting A3A from degradation similar to high-risk E7-expressing cells. CUL2 was shown to interact with both A3A and E7, suggesting CUL2 as a key player in preventing A3A degradation. Using an in vitro cytidine deaminase assay, they showed that the A3A protein stabilized by HPV16 E7 maintained its enzymatic activity to deaminate cytidine. These findings that high-risk HPV E7 stabilizes A3A protein provide a new insight into virus-induced mutagenesis that may contribute to the generation of APOBEC3 mutation signatures observed in HPV-positive cancers.

**Emily Anne Kizer** (University of Idaho, ID, USA) discussed the identification of novel antibiotics from satellite viruses. Increasing levels of drug resistance in human pathogenic yeasts stress the need for to develop new antifungal drugs. The Kizer lab has screened 216 yeasts from the *Saccharomyces* genus and identified 30 strains capable of producing protein toxins that inhibit the growth of fungi, including other *Saccharomyces* yeasts and several human pathogens from the genus *Candida*. They have found that 77% of these newly identified “killer yeasts” were infected with viruses from the *Totiviridae* family. Of these virus-infected killer yeasts, 93% contained double-stranded RNA (dsRNA) satellites, which have been previously shown to encode antifungal toxins. The molecular weight and copy number of these dsRNA satellites varies within each killer yeast. The antifungal toxins produced by each identified killer yeast have the ability to inhibit growth of a unique subset of *Saccharomyces* and pathogenic yeast strains. They hypothesized that sequence polymorphisms found within dsRNA satellites were responsible for the variation in toxin tropism. To explore the relationship between genotype and antifungal toxin efficacy, they developed a rapid method for dsRNA extraction and next-generation sequencing. Using this pipeline, they have successfully identified several novel totiviruses and toxin-encoding dsRNA satellites. The expressed cloned toxin genes from dsRNAs in the laboratory yeast *S. cerevisiae*, recapitulating their antifungal activity. This was the first study to investigate the role of dsRNA satellites in the production of antifungal toxins and to relate dsRNA satellite sequence to the killer yeast phenotype.

### 2.2. DNA Viruses

**Keith W. Jarosinski** (Department of Pathobiology, College of Veterinary Medicine, University of Illinois at Urbana-Champaign, Champaign, IL, USA) described his lab’s studies to identify host-specificity during herpesvirus host-to-host transmission, with the overall goal of understanding the mechanism by which viral and host proteins facilitate efficient transmission from host-to-host. Transmission from host-to-host (interindividual spread) is an essential component in the herpesvirus’ life cycle. Herpesviruses are typically associated with a single host species in nature, but when they transmit across species (interspecies spread), the outcome is often deadly. For example, the macaque herpes B virus causes fatal encephalitis when transmitted to humans, and Aujeszky’s disease in cattle is caused by Suid alphaherpesvirus 1 (SuHV1), better known as pseudorabies virus (PRV), naturally found in pigs. However, very little is known about transmission of herpesviruses. He described his lab’s successful work that culminated in identification of two herpesvirus proteins that are essential for interindividual spread of Gallid alphaherpesvirus 2 (GaHV-2), better known as Marek’s disease virus (MDV), in chickens-namely, glycoprotein C (gC) that is conserved among the Alphaherpesvirinae subfamily and the conserved herpesvirus protein kinase (CHPK) conserved among all members of the Herpesviridae family.

**Myrna M. Miller** (University of Wyoming, Wyoming State Vet Lab, Laramie, WY, USA) continued the theme of herpesviruses in natural hosts by presenting her group’s study of seroprevalence of a novel mule deer-associated alphaherpesvirus in Wyoming. A previously unidentified alphaherpesvirus was found in juvenile mule deer (*Odocoileus hemionus*) that were submitted to the Wyoming State Veterinary Laboratory with infectious keratoconjunctivitis-like syndrome (IKC) and/or blindness. A retrospective analysis of IKC cases between 2000 and 2016 in mule deer found that 23% (4/17) were associated with this herpesvirus. Whole-genome sequencing of four isolates found that they were identical (>99.8% identity). Phylogenic analysis based on amino acid sequences for glycoproteins gB, gC, and gD showed this mule deer-derived herpesvirus (MDHV) is most closely related to ruminant alphaherpesviruses, including cervid herpesvirus-1 (CvHV-1, 93% identity), cervid herpesvirus 2 and bovine herpesvirus 1 (CvHV-2, BHV-1, 91% identity), BHV-5 and buffalo herpesvirus (90% identity), and caprine herpesvirus (86% identity). BHV-1 is the prototype for ruminant alphaherpesviruses and causes respiratory and reproductive syndromes in cattle, including rhinotracheitis, conjunctivitis, encephalitis, and abortion. The distribution and prevalence, as well as the range of clinical syndromes caused by MDHV, are currently unknown. A seroepidemiologic survey was performed to establish the prevalence of MDHV infection in three Wyoming herd units. Microtiter serum neutralization assays were used with mule deer serum against MDHV or BHV-1. The prevalence of anti-MDHV antibodies in these three herds was 84% (67/80, CI 90%, 77–91%), 90% (66/73, CI 90%, 84–96%), and 76% (68/89, CL 90%, 69–83%). Serologic cross-reactions with BHV-1 were detected, but titers were significantly lower (*p* values </= 0.008) based on paired students *t*-test. MDHV is highly prevalent in Wyoming mule deer and cross-reacts with BHV-1 at significantly lower titers.

**Linda F. van Dyk** (Department of Immunology and Microbiology, University of Colorado School of Medicine, Aurora, CO, USA) presented her lab’s work using a mouse model to study human herpesvirus disease. Gammaherpesviruses (γHV), including Epstein-Barr virus, Kaposi’s sarcoma associated herpesvirus (KSHV) and murine γHV68, exploit lymphocytes as their lifelong reservoir. Previously, the van Dyk lab demonstrated that a conserved viral homolog of cellular cyclins (v-cyclin) is critical for specific aspects of γHV68 infection. The v-cyclin is critically required for reactivation from latency, viral persistence, and disease progression, including tumor development, yet is dispensable for viral replication and establishment of latency. They found that v-cyclin function in reactivation was complemented by the v-cyclin of KSHV and by host cyclin D3, but not by other related cyclins. Additionally, they found that the v-cyclin promoted reactivation from latency by specific antagonism of host cyclin dependent kinase inhibitor, p18INK4C (p18). Infection of p18 null mice with a v-cyclin deficient virus (cycKO) restored reactivation from latency to near wild-type virus levels, and using a recombinant γHV68 virus that expressed host p18 in place of the v-cyclin (p18KI) resulted in significantly suppressed reactivation beyond that of the cycKO virus. The cyclin D/p18 regulatory hub was critically required for normal B cell development and differentiation, and was disrupted in many B cell lymphomas. This research further characterizes the specific interplay between distinct cyclin functions and host p18 in virus reactivation, and illustrates the therapeutic potential of targeting host p18 to manipulate virus infection and associated disease.

**Elizabeth A. Fortunato** (Department of Biological Sciences and Center for Reproductive Biology, University of Idaho, Moscow, Idaho, USA) continued the theme of host-virus interactions and disease caused by herpesvirus infection by presenting her work showing that human cytomegalovirus (HCMV) controls cellular nidogen 1 protein levels and its implication on the developing fetus. HCMV congenital infection in the US affects ~1% of newborns annually. Five to 10% of congenitally infected infants display negative central nervous system consequences at birth and another 10–15% develop sequelae within the next few years, principally sensorineural hearing loss. Malfunctioning of the nervous system later in childhood indicates a prolonged viral interaction or propagation of a deficiency. Her lab’s observation of infection-induced DNA damage to chromosome 1, at 1q23 and 1q42, indicated distinct interactions of HCMV with the cellular DNA. Fine-mapping of the 1q42 breaksite revealed Nidogen 1 (NID1), an important basement membrane protein, was encoded in the region. NID1 is expressed by many cells within the developing brain and has been shown to be important for neuronal migration, optic cup morphogenesis and neural network excitability and plasticity. Downregulation of NID1 expression could have negative ramifications for an infected fetus. They now find that NID1 is downregulated not only transcriptionally, but also at the post-translational level. pp71 is bound to the DNA at the 1q42 breaksite and the NID1 promoter leading her to suspect it, in conjunction with the insulator protein CTCF, is responsible for transcriptional regulation. NID1 protein levels are also decreased after Δpp71 virus infection. The targeting of NID1 by two separate viral proteins via two separate pathways highlights the importance of NID1’s elimination to the virus. She concluded by describing her future plans that will focus on characterizing the NID1 targeting mechanisms, defining the benefits of NID1 downregulation to viral infection, and defining the negative ramifications of NID1 downregulation to an infected fetus.

**Teresa Mescher** (Department of Neurology, University of Colorado School of Medicine, Aurora, CO, USA) described work providing a mechanistic pathway through which the neurotropic alphaherpesvirus, varicella zoster virus, may contribute to the development of a serious disease in the aging human population. Giant cell arteritis (GCA) is an inflammatory vascular disease of the elderly that has been shown to be highly associated with varicella zoster virus (VZV) infection of the temporal artery (TA) [1,2,3]. GCA is pathologically defined as having transmural inflammation, medial necrosis, and giant and/or epithelioid cells. Interleukin (IL)-8 is an inflammatory cytokine that when released, calls in neutrophils to the site of an infection. IL-8 is upregulated in the cerebral spinal fluid (CSF) of patients with VZV vasculopathy [4], and in VZV-infected primary human vascular cells in vitro [5]. Based on these findings and on the presence of abundant VZV antigen in GCA, 20 GCA-positive (patients with clinical and laboratory features of GCA whose TA biopsies are pathologically positive for GCA), 20 GCA-negative (patients with clinical and laboratory features of GCA whose TA biopsies are pathologically negative for GCA), and 18 normal TAs were stained for the presence of IL-8 and neutrophils (CD15). Immunohistochemical analysis revealed interleukin 8 (IL-8) antigen in 17/20 (85%) GCA-positive, 18/20 (90%) GCA-negative, and 2/18 (11%) normal TAs. CD15 antigen was also seen in 20/20 (100%) GCA-positive, 18/20 (90%) GCA-negative, and 3/18 (17%) normal TAs. IL-8 appears to be upregulated in both GCA-positive and -negative TAs compared to normal TAs. The upregulation of IL8 is also consistent with the presence of neutrophils in all GCA-positive TAs and most GCA-negative TAs. This finding further shows that GCA is an immunopathological disease caused by VZV reactivation in the cranial ganglia and subsequent infection of the TA.

**Christina Como** (University of Colorado School of Medicine, Aurora, CO, USA) presented her progress in developing and in vitro model of varicella zoster virus latency. Varicella zoster virus (VZV) is an alphaherpesvirus that causes varicella (chicken pox) and establishes latency in sensory neurons along the entire human neuraxis. Virus reactivation is commonly observed in the elderly and immunosuppressed individuals and results typically in shingles, but can also cause postherpetic neuralgia, vasculopathy, and ocular diseases. Although there is a vaccine to mitigate varicella, the vaccine is a live attenuated virus that can still enter latency and reactivate to cause disease. Currently, there is no animal model available to study VZV, making it important to design a cell model for latency and reactivation. It is hypothesized that a persistent release of cytokines suppresses virus and contributes to latency in human neurons. We hypothesize that VZV-infected neurons treated with cytokines will establish latency in vitro. Our results suggest interleukin 6 (IL-6), tumor necrosis factor (TNF)-α, interferon alpha (IFNα), and IFNβ individually aid in limiting VZV growth and spread. This provides a better understanding of the relationship between the immune system, human neurons, and VZV latency. Using this model and understanding these interactions may lead to treatment options for patients experiencing VZV pathology.

**Nicholas Baird** (Departments of Neurology University of Colorado School of Medicine, Aurora, CO, USA) expanded on the in vitro neuronal culture model to develop a system to investigate the molecular events involved in virus-induced pain. Varicella zoster virus, an alphaherpesvirus, infects and establishes latency in sensory, autonomic, sympathetic and parasympathetic neurons during primary infection (varicella; chickenpox). Decades later, Varicella zoster virus (VZV) can reactivate to cause a multitude of neurological disorders including zoster (shingles) and post-herpetic neuralgia (PHN), pain lasting months to years following resolution of zosteriform rash. His model for virus-induced pain involved differentiating a human dorsal root ganglion cell line, HD10.6 into nociceptive sensory neurons which express βIII-tubulin, peripherin and sodium channels 1.7 & 1.8. Importantly, the differentiated cells express substance P and NK1, a molecule and receptor involved in signaling and sensing nociceptive pain. These cells were permissive to VZV infection, that culminated in the production of infectious virus. Together, this new model system is poised to begin understanding the molecular mechanisms underpinning the pains of zoster and PHN.

**Anna Blackmon** (University of Colorado, School of Medicine, Aurora, CO, USA) presented data describing studies to investigate how a nehrotorpic alphaherpesvirus could exit the neuron during virus reactivation. Varicella zoster virus (VZV) vasculopathy occurs after virus reactivates from ganglia, spreads along nerve fibers to arteries, leading to pathological vascular remodeling and stroke. An interesting observation in temporal arteries from VZV vasculopathy patients was the presence of VZV antigen in cells surrounding nerve fibers in the outermost adventitial layer. Immunohistochemistry revealed that these VZV-infected cells expressed the tight junction protein claudin-1, identifying them as perineurial cells which form a barrier between the peripheral nerve and surrounding tissue. Her lab hypothesized that during VZV spread along nerve fibers, the virus may disrupt tight junctions in perineurial cells, potentiating infection of surrounding vascular cells. To test this hypothesis, they mock- and VZV-infected primary human perineurial cells (HPNCs) and compared expression and distribution of cell adhesion proteins (claudin-1, E-cadherin and N-cadherin) at three days post-infection. While there was no VZV-induced changes in claudin-1 transcripts compared to mock-infected HPNCs, claudin-1 redistributed from the membrane/cytoplasm to the nucleus. When HPNCs were treated with anti-claudin-1 or isotype control antibodies prior to VZV-infection, there was no difference in the level of virus infection. Furthermore, while mock-infected cells expressed E-cadherin and not N-cadherin, VZV-infected cells did not express E-cadherin but expressed N-cadherin-supporting the novel possibility of a VZV-induced epithelial-mesenchymal cell transition. Addition of conditioned media from VZV- or mock-infected HPNCs to uninfected HPNCs showed that the conditioned media from VZV-infected cells contained a soluble factor that was able to induce the same changes seen in VZV-infected cells. Overall, their findings suggest that: (1) claudin-1 is not necessary for VZV infection and (2) VZV-induced redistribution of claudin-1, downregulation of E-cadherin and upregulation of N-cadherin is mediated by a soluble factor and may lead to disruption of tight junctions in perineurial cells, allowing viral spread from nerve fibers to surrounding vascular cells in VZV vasculopathy.

**Catherine Pearce** (Department of Neurology, University of Colorado School of Medicine, Aurora, CO, USA) presented a study describing how varicella zoster virus alters lipid metabolism in human sensory neurons and permissive human lung fibroblast cells. Varicella zoster virus (VZV) is an alphaherpesvirus that upon primary infection causes a dermatomal rash in skin fibroblasts, after which it becomes latent in ganglionic neurons. Statins have been a major advancement in the prevention of cardiovascular disease; however, patients who take this medication to reduce cholesterol levels appear to experience an increased risk of VZV reactivation, the cause of shingles. How statins and cholesterol levels influence VZV reactivation in latently-infected ganglionic neurons is unknown. To better understand the relationship between cholesterol metabolism and VZV infection in neurons and fibroblasts, The Baird lab performed a microarray-based gene analysis of lipoprotein and cholesterol metabolism pathways. Their results showed that VZV infection modulates expression of genes involved in cholesterol metabolism and lipoprotein signaling in both human neurons and fibroblast cells. Further studies were described to help elucidate mechanisms of how VZV-infection may alter lipid metabolism for a better understanding of why patients on statins are more likely to experience zoster.

**Claire Birkenheuer** (Louisiana State University, Baton Rouge, LA, USA) showed high resolution mapping of host RNA polymerase II following herpes simplex virus type 1 infection, to more clearly understand how the virus repatriates the enzyme for viral transcription. Precision nuclear run-on (Pro-seq) is used to map RNA Polymerase II (Pol II) location on actively transcribed genomes to a single base pair resolution. Using this technique, the Baines’ lab characterized Pol II occupancy on host genes 3 h post-infection with herpes simplex virus 1 (HSV-1). Their data shows that even at this early time point Pol II is removed from most host genes. However, they also noted gene-specific effects in which HSV-1 increases Pol II occupancy and modulates its location at every stage of transcription: from Pol II recruitment and initiation, to promoter proximal pausing, elongation, and termination. To assess the effects of this modulation, the Baines’ lab used quantitative reverse transcription PCR (qRT-PCR) with total RNA, nuclear messenger RNA (mRNA), and cytoplasmic mRNA to analyze transcript levels of STUB1, USP8, MYC, EGR1, FOSB, c-FOS, and JUNB. Most genes with increased Pol II occupancy or recruitment had higher levels of total RNA and nuclear mRNA. In contrast, termination extension usually decreased cytoplasmic mRNA levels. Functional clustering revealed a profile that, they hypothesize, reflects a cellular environment permissible for virus replication. Thus, HSV-1 reduces host gene expression, working in combination with the virion host shut-off protein (vhs) in some cases, while ramping up host gene transcription to transcend the effects of vhs in other cases. They note that Pro-seq overcomes technical difficulties associated with Pol II chromatin immune precipitation and sequencing while providing higher resolution, and holds promise for use with other viruses that alter Pol II activity.

**Addilynn Beach** (University of Colorado School of Medicine, Denver, CO, USA) also investigated virus gene transcript, but at the level of transcription initiation. Specifically, she searched for chromatin insulator binding on the virus genome during latency to determine how the virus genes are silenced in human neurons. Herpes simplex virus type 1 and varicella zoster virus are ubiquitous human neurotropic alphaherpesviruses that establish life-long latent infection in trigeminal ganglia following primary infection. During latency, most virus genes are transcriptionally silenced by posttranscriptional modifications of bound histone proteins. Current thought suggests that the epigenetic markings on the latent virus genome are segregated by chromatin insulators, especially the CTCF-binding factor (CTCF). To develop a topological profile of alphaherpesviruses genome in multiple human trigeminal ganglia, a set of tilled oligonucleotides were used to enrich virus sequences in chromatin immunoprecipitation assays targeting CTCF-protein. Analysis of human DNA within the enriched sample described definite peaks at known CTCF-motifs located at cell gene promoters. Analysis of virus DNA within the samples showed read clusters enriched at CTCF-motifs. It is hoped that our consensus portrait of CTCF occupancy on alphaherpesviruses in human trigeminal ganglia will provide clues to the mechanism by which virus gene transcription is controlled during latency and reactivation.

### 2.3. Prion Disease

**Thomas E. Eckland** (Medical Microbiology and Immunology, Creighton University in Omaha, NE, USA) introduced prion disease, and how prion strain interactions during incubation periods affect strain dynamics. Prion diseases are a group of inevitably fatal neurodegenerative diseases affecting mammals, including humans. Prion diseases are caused by a misfolded isoform of the prion protein, PrP^Sc^. Prion strains are operationally defined a heritable phenotype of disease under controlled transmission conditions that are hypothesized to be encoded by strain-specific conformations of PrP^Sc^. Prion strains can interfere with each other when a long incubation period strain (i.e., blocking strain) inhibits the replication of a short incubation period strain (i.e., non-blocking). Prion strain interference influences prion strain dynamics and the emergence of a strain from a mixture. However, it is unknown if two long incubation period strains can interfere with each other. To investigate this, they used a combination of protein misfolding cyclic amplification strain interference (PMCAsi) and animal bioassay. Using PMCAsi, they found a PrP^Sc^ migration pattern consistent of a mixture of both strains, suggesting that strain interference is not occurring. Animal bioassay experiments are consistent with these in vitro findings. This is the first example of prion strain combinations that do not interfere with each other both in vitro and in vivo. This observation changes the paradigm of the interactions of prion strains in a mixture and has implication for interspecies transmission and emergence of prion strains from a mixture.

**Kaitlyn Miedema** (Department of Microbiology, Immunology and Pathology, Colorado State University in Fort Collins, CO, USA) described the role of plants in transmission of chronic wasting disease (CWD). Recent work has found both internal and external plant surfaces are able to bind and retain infectious prions, suggesting plants may play an important role in prion transmission. To expand on these findings, the Zabel lab exposed rice plants (*Oryza sativa* L., cultivar *kitaake*) to a solution containing 1% of a brain homogenate from a terminally ill elk for either 2 h, 24 h or 72 h. Tissues were collected by cutting the leaves, stem and roots and were then homogenized to a 10% (*w*/*v*) homogenate and tested for chronic wasting disease prions (PrP^CWD^) by protein misfolding cyclic amplification (PMCA). After PMCA, we were able to detect PrP^CWD^ in root and leaf tissue of experimentally exposed rice plants by 2 h post exposure (hpe). At 24 hpe, PrP^CWD^ was detected in root tissues after PMCA. No positivity was detected by PMCA at 72 hpe. In addition to laboratory manipulations, we wanted to investigate whether plants were contaminated with PrP^CWD^ in a natural setting. Two sites were identified in Rocky Mountain National Park (RMNP) and plants were collected using disposable spades. Plant surfaces were washed and wash solution was tested for PrP^CWD^ by PMCA. After washing and decontamination, plants were homogenized to test for the presence of PrP^CWD^ on internal structures. We detected PrP^CWD^ from the surface of two plants at a single location in RMNP after six rounds of PMCA. None of the internal tissues were positive by PMCA. These results provide new insights into the role of plants in CWD transmission.

**Erin McNulty** (Department of Microbiology, Immunology and Pathology, Colorado State University in Fort Collins, CO, USA) presented progress on in vitro prion detection methodology throughout clinical prion disease. Prion infectivity can be transmitted in blood during symptomatic and asymptomatic disease phases of Creutzfeldt-Jakob disease (vCJD)-infected humans, and chronic wasting disease (CWD), bovine spongiform encephalopathy (BSE) and scrapie-infected animals. It is currently estimated that ~1:2000 individuals in the United Kingdom are asymptomatic carriers of vCJD. CWD continues to demonstrate geographic expansion, now found in captive and free-range cervid populations in North America, South Korea, and Norway. Progress in developing in vitro detection assays for hematogenous prions has been hampered by the presence of inhibitors found in blood that interfere with detection methodologies. The development of antemortem detection of blood-borne prions for humans, cervids and other susceptible species is of utmost importance for human public health, animal monitoring of prions, and improved understanding of intra/inter-host trafficking and pathogenesis. Their previous work demonstrated CWD prion infectivity and an ability to detect prions by in vitro methodology in blood throughout the protracted disease course. Here they corroborated and improved upon these findings by use of blood buffy coat cells and superparamagnetic iron oxide beads (IOMB) in combination with real time quaking-induced conversion (RT-QuIC). They have demonstrated prions by IOMB-RT-QuIC in blood harvested minutes post prion exposure (15 min, 30 min, 60 min, one week, two weeks, *n* = 8 deer with 16 replicates/animal/time) through clinical disease (*n* = 7 deer, with 24–36 replicates/animal). This methodology will: (i) enhance our ability to determine the role of blood-borne prions in establishing and maintaining infection; as well as (ii) identify which specific blood cell phenotype subsets traffic/amplify prions through the disease course.

**Candace Mathiason** (Colorado State University, Fort Collins, CO, USA) continued the theme of pathogen and host interactions through a presentation of their use of real time quaking-induced conversion (RT-QuIC) to detect blood-borne prions in buffy coat cells harvested from longitudinal chronic wasting disease (CWD) infection studies. Prion infectivity has been transmitted in blood during symptomatic and asymptomatic disease phases of Creutzfeldt-Jakob disease (vCJD)-infected humans, and CWD, bovine spongiform encephalopathy (BSE), and scrapie-infected animals. It is currently estimated that ~1:2000 individuals in the United Kingdom are asymptomatic carriers of vCJD. CWD continues to demonstrate geographic expansion, now found in captive and free-range cervid populations in North America, South Korea, and Norway. Progress in developing in vitro detection assays for hematogenous prions has been hampered by the presence of inhibitors found in blood that interfere with detection methodologies. The development of antemortem detection of blood-borne prions for humans, cervids and other susceptible species is of utmost importance for human public health, animal monitoring of prions, and improved understanding of intra/inter-host trafficking and pathogenesis. Their previous work demonstrated CWD prion infectivity and an ability to detect prions by in vitro methodology in blood throughout the protracted disease course. Here they corroborated and improved upon these findings by use of blood buffy coat cells and superparamagnetic iron oxide beads (IOMB) in combination with RT-QuIC. They demonstrated prions by IOMB-RT-QuIC in blood harvested minutes post prion exposure (15 min, 30 min, 60 min, 1 week, 2 weeks, *n* = 8 deer with 16 replicates/animal/time) through clinical disease (*n* = 7 deer, with 24–36 replicates/animal). This methodology will: (i) enhance our ability to determine the role of blood-borne prions in establishing and maintaining infection; as well as (ii) identify which specific blood cell phenotype subsets traffic/amplify prions through the disease course.

**Byron Caughey** (Rocky Mountain Laboratories, NIAID, NIH, in Hamilton, MT, USA) described ultra-sensitive assays for prions and prion-like misfolded protein aggregates. Many neurodegenerative diseases involve the pathological accumulation of fibrillar aggregates of specific proteins such as Aβ and tau in Alzheimer’s disease; tau in frontotemporal dementias, chronic traumatic encephalopathy and other tauopathies; αSynuclein in Parkinson’s and other synucleinopathies; and prion protein (PrP) in prion diseases. In these diseases, the protein aggregates propagate within, and sometimes between, hosts through seeded polymerization mechanisms. A major clinical problem is the inability to accurately discriminate and diagnose many of these diseases, which can have complex and overlapping clinical manifestations, while patients are still alive. We and others are exploiting the basic propagation mechanisms of various misfolded protein aggregates in vitro to develop accurate antemortem diagnostic tests based on etiological biomarkers. Multiple large independent studies have now shown 92–100% sensitivity and nearly 100% specificity of second generation (IQ-CSF) RT-QuIC assays in the diagnosis of sporadic Creutzfeldt-Jakob disease using cerebrospinal fluid and/or nasal swab samples. RT-QuIC assays for most other prion diseases of humans and animals have also been developed and are undergoing further evaluation. Analogous prototypic RT-QuIC and protein misfolding cyclic amplification (PMCA) assays for tauopathies and synucleinopathies have also begun to appear. For example, they have developed a tau RT-QuIC assay that can detect ~billion-fold dilutions of brain tissue from human cases with a type of tauopathy (Pick’s disease) with a high degree of sensitivity and specificity relative to other types of tauopathies. Collectively these seeded fibrillization assays for disease-specific and pathological protein aggregate biomarkers have the potential to greatly improve the development of diagnostics and therapeutics for multiple neurodegenerative protein misfolding diseases.

### 2.4. RNA Control

**Richard E. Lloyd** (Department of Molecular Virology and Microbiology, Baylor College of Medicine, Houston, TX, USA) described RNA granules and how these unique cytoplasmic structures may cross-talk with the innate immunity system. Two types of dynamic cytoplasmic RNA granules are conserved from yeast to mammals. Stress granules (SGs) contain stalled translation initiation complexes, and processing bodies (PBs) are enriched with RNA decay factors. Since both types of RNA granules sequester translationally silenced mRNAs, RNA viruses antagonize RNA granules for replicative advantages. Enteroviruses and other picornaviruses repress SG and PB formation via multiple mechanisms, however, enteroviruses principally block SG through cleavage of the dominant SG-nucleating factor Ras GTPase-activating protein-binding protein 1 (G3BP1). The Lloyd lab has shown that G3BP1 functions as an activator of innate immunity via stress-induced recruitment of PKR to SGs and also activates NF-κB. In addition, G3BP1 undergoes reversible phosphorylation and methylation changes during stress and recovery which are required for G3BP1 to nucleate stress granules, and which regulate innate immune activation. These findings support an emerging concept that SGs function as signaling platforms to activate or enhance innate immunity and better define the antagonistic relationship of viruses with RNA granules.

**Nicholas Meyerson** (BioFrontiers Institute, University of Colorado, Boulder, CO, USA) described how cellular Tripartite motif-containing protein 25 (TRIM25) senses and restricts the Influenza A Virus ribonucleoprotein complex. The human genome encodes approximately 100 TRIM E3 ubiquitin ligases. Although many TRIMs have been linked to viral infection, detailed mechanisms are known for only a small number. TRIM25 is a component of the RIG-I signaling cascade that activates the interferon response upon sensing foreign viral RNA. The Meyerson lab described a novel function of TRIM25 whereby it inhibited influenza viral RNA synthesis in a direct manner, which was independent of its ubiquitin ligase activity and of the interferon response in general. Therefore TRIM25 is a bona fide restriction factor that blocks virus replication directly without the need for extensive signaling. The mechanism by which TRIM25 inhibits viral RNA synthesis is unique. TRIM25 senses and binds vRNPs, the influenza structures which contain viral genome segments. Their results provide strong evidence that TRIM25 acts as a molecular clamp that impedes movement of the viral RNA template into the polymerase. By this mechanism TRIM25 does not directly inhibit polymerase activity, but rather deprives the polymerase of access to the viral RNA template. Furthermore, they find that the restriction factor activity of TRIM25 is specific to influenza viruses and is antagonized by the influenza virus accessory protein NS1. They have also shown that TRIM25 has evolved under positive selection in both mammalian and avian species that are regularly infected with influenza. This unique evolutionary signature results from recurrent antagonism by viruses and suggests that TRIM25 may act as a barrier to the cross-species transmission of influenza viruses in the wild.

**Justin Lee** (Colorado State University, Fort Collins, CO, USA) described new advances in next-generation nucleic acid sequencing through the introduction of MinION; a platform especially useful for assembling RNA virus genomes in remote research stations. The MinION by Oxford Nanopore Technologies Inc. is the first portable next-generation sequencing (NGS) instrument and is capable of producing long-reads of single molecules from DNA and RNA libraries. The NGS Facility in the Department of Microbiology, Immunology and Pathology at Colorado State University has a MinION instrument and has recently performed pilot testing of this instrument to better understand its capabilities and limitations. They obtained sequence data from bacterial and eukaryotic genomic DNA on the MinION and on an Illumina MiSeq. The MinION runs produced reads ranging from less than 1 kb to over 500 kb in length with an error rate of approximately 25%. Output from a single run was sufficient to assemble a complete draft 6 Mbp bacterial genome sequence in one contig, with adequate depth to minimize the effect of the high error rate. A hybrid assembly incorporating long and short reads produced a near perfect assembly of the bacterial genome. Output from a single MinION run produced only ~0.1× coverage over a bat genome, and thus was not sufficient to assemble the genome. They conclude that the MinION is a valuable tool for small genome sequencing and for producing long reads to span areas of low complexity that don’t assemble well with short-read data alone. The low upfront cost and portability of the instrument also make it a possibly useful tool for field and surveillance applications.

**Benjamin M. Akiyama** (Department of Biochemistry and Molecular Genetics, University of Colorado Denver School of Medicine, Aurora, CO, USA) described nuclease resistant non-coding RNA in Zika virus. The outbreak of Zika virus (ZIKV) and associated fetal microcephaly mandates efforts to understand the molecular processes of infection by this emerging global pathogen. Related flaviviruses, including Dengue and West Nile virus, produce unique non-coding RNAs known as subgenomic flaviviral RNAs (sfRNAs). sfRNAs are highly conserved and have been linked to the pathology of the virus in cell culture and mouse model systems. sfRNAs are formed by co-opting Xrn1, the cell’s predominant 5′→3′ exonuclease. The viral RNA genome contains highly structured Xrn1-resistant RNAs (xrRNAs), which halt the enzyme leaving sfRNAs behind. While the biology of sfRNAs has been explored in other flaviviruses, the existence of sfRNAs or xrRNAs during ZIKV infection has not been reported. The Akiyama lab demonstrated that ZIKV infection in multiple cell types results in distinct sfRNA patterns. They solved the structure of a ZIKV xrRNA responsible for sfRNA production in these cell types. The complete ZIKV xrRNA structure reveals how xrRNAs resist the helicase activity of Xrn1, using two intertwined RNA pseudoknots which form a molecular “slipknot.” Furthermore, the ZIKV xrRNA structure provides a model for understanding Xrn1-xrRNA interactions, and identifies conserved features that modulate function in diverse pathogenic flaviviruses.

**Colleen L. Watkins** (Department of Biochemistry and Molecular Biology, Colorado State University, Fort Collins, CO, USA) described the role of picornaviral polymerase finger domains in RNA binding and translocation. The picornavirus family of viruses includes poliovirus, the causative agent of paralytic polio and coxsackievirus, which is responsible for viral-heart-disease. Picornaviruses contain a single-stranded positive-sense RNA genome replicated by 3 Dpol, an RNA-dependent RNA polymerase (RdRP). Crystal structures of 3Dpol from multiple picornaviruses have shown a conserved polymerase fold analogous to a “right hand” composed of fingers, palm and thumb domains. A previous study in coxsackievirus showed mutations within the RdRP palm domain resulted in changes to both fidelity and elongation rate, with slower replicating RdRPs having a higher fidelity and lower fidelity RdRPs replicating faster [6]. Mutations in the fingers domain primarily changed the rate with less of an effect on fidelity. This finding led to the hypothesis that regulation of speed and fidelity are controlled by different regions within the RdRP, with the rate of translocation being controlled by the fingers domain as an entire unit. To address this, a set of chimeric polymerases based on poliovirus and coxsackievirus 3Dpols have been constructed by exchanging the fingers domains entirely or only the pinky finger motif between the two viral polymerases, and the hybrid 3 Dpols were characterized. The data suggest determinants of RNA binding are contained within the polymerase pinky finger, while elongation complex stability is established by the polymerase core. Significant effects to translocation are not seen, likely due to active site closure by the wild type polymerase cores remaining the rate limiting step in the catalytic cycle.

**Anna-Lena Steckelberg** (University of Colorado, Denver, CO, USA) showed how viral RNA was protected from 5′–3′ exoribonucleolytic decay by enzyme-assisted RNA folding. RNA viruses generally have short genomes and encode only a few proteins, thus they have evolved elegant RNA-based strategies to manipulate cellular processes and thereby enhance virus propagation. An interesting illustrative example is found in plant-infecting Dianthoviruses, which generate viral non-coding RNAs through the selective protection of their 3′ untranslated region (UTR) from 5′–3′ exonucleolytic decay. The Steckelberg lab discovered that a 44-nucleotide structured RNA element at the beginning of the viral 3′ UTR was sufficient to inhibit 5′–3′ exonucleolytic decay in vitro. They solved the three-dimensional structure of a complete Dianthovirus nuclease-resistant RNA (xrRNA) by X-ray crystallography, revealing that it forms a stem loop structure which is held in a distorted “tilted” conformation through several long-distance base-stacking interactions. Base pairs formed between adjacent molecules in the crystal structure indicate that this Dianthovirus xrRNA can adopt two mutually exclusive conformations, and functional assays with mutant RNAs confirm that both conformations are required for nuclease resistance. These data suggest that the xrRNA element functions as a structural switch, triggered by the arrival of an exonuclease from the 5′ direction. Whereas the tilted stem-loop structure likely represents a necessary folding intermediate, the actual nuclease-resistant structure contains a pseudoknot formed between the 3′ end of the structure and the loop sequence, which generates a ring-like fold that protectively wraps around the 5′ end of the structure. A similar ring-like shield around the 5′ end of RNA is found in the nuclease-resistant RNA structures of arthropod-borne flaviviruses (such as Dengue and Zika virus); although the folding strategies used to form the two structures are very different. Even though the overall fold of flaviviral and dianthoviral nuclease-resistant RNAs differs greatly, this observation highlights how highly divergent RNA viruses have developed similar RNA-based strategies to inhibit exoribonucleases, and suggests that manipulation of nuclease activity through RNA structure might be a widespread mechanism.

**Zoe G. O’Donoghue** (Department of Biochemistry & Molecular Genetics, University of Colorado Anschutz Medical Campus, Aurora, CO, USA) explored and characterized the pathogenically relevant RNA structures in Flaviviruses. Flaviviruses like Dengue, Zika, and West Nile infect millions of people every year making them prominent global health threats. Broadly speaking, the family flaviviridae contains enveloped viruses that are vector borne and have positive sense, single stranded RNA genomes. Throughout infection viral replication produces large quantities of genomic RNA, but the accumulation of smaller regions of the highly structured viral 3′ untranslated region (UTR) is also observed. These subgenomic Flaviviral RNAs (sfRNAs) are made via incomplete degradation of the viral genome by the host 5′ to 3′ exonuclease Xrn1, and previous studies show that sfRNA formation is dependent upon the presence of highly structured and conserved regions of the 3′ UTR called Xrn1 resistant RNAs (xrRNAs). sfRNAs have been shown to be necessary for both cytopathicity and pathogenicity during West Nile virus (WNV) infection. Using a reconstituted in vitro study system, the Kieft lab has demonstrated that several alternate members of the flaviviridae family, including Modoc (MODV), Montana Myotis Leukoencephalitis (MMLV), Cell Fusing Agent (CFAV), and Tick Borne Encephalitis (TBEV) viruses, also produce sfRNAs following Xrn1 degradation. Further, using selective 2′ hydroxyl acylation analyzed by primer extension (SHAPE) chemical probing they have been able to characterize these evolutionarily diverse xrRNAs into two distinct structural “classes”, indicating that while the ability to produce sfRNAs may be conserved across the flavivirus genus, the sequences required for Xrn1 halting are more variable than previously suspected. This observation may imply a generalized role for sfRNAs during flavivirus infections and currently they are working to more fully characterize the structural mechanism(s) of enzyme halting in these different classes of xrRNAs. Additionally, they have found that the halt sites in the 3′ UTR of WNV are capable of stopping other exonucleases as well, including bacterial enzyme RNase J1. Ongoing studies also include infection models and large-scale manipulations of xrRNAs in the Dengue Virus 3′UTR to explore the structure-function mechanisms of sfRNAs during infection in both mosquito vector and vertebrate host models.

**Tony Schountz** (Department of Microbiology, Immunology and Pathology, College of Veterinary Medicine and Biomedical Sciences, Colorado State University, Fort Collins, CO, USA) presented information concerning the experimental infection of Jamaican Fruit Bats (*Artibeus jamaicensis*) with a rescued bat HL18NL11 Influenza A-like virus. Recently, nucleotide sequencing led to the discovery of the presence of two novel influenza A-like viruses, HL17NL10 and HL18NL11, in New World little yellow shouldered fruit bats (*Sturnira lilium*) and flat-faced fruit bats (*Artibeus planirostris*), respectively. Serological studies indicated high prevalence to these viruses among many species of Phyllostomidae leaf-nosed fruit bats of Central and South America, including Jamaican fruit bats (*Artibeus jamaicensis*). Infectious viruses have not been isolated from bats: therefore an infectious clone of HL18NL11 was generated by reverse genetics technologies that produced particles resembling influenza viruses from transfected cells by electron microscopy. The Schountz lab sought to determine susceptibility of Jamaican fruit bats to rescued HL18NL11 bat influenza A-like virus. During a 28 day challenge experiment with intranasal inoculation, the bats exhibited no clinical signs of disease. However, rectal swabs had up to 104 TCID50 equivalents of HL18NL11 viral RNA (vRNA) by real-time PCR in each bat on days 2, 4 and 7 post-inoculation, and in the lungs of one of the bats on day 28 when they were euthanized. Histopathology revealed minimal evidence of disease except for the one bat with detectable vRNA in its lung. This bat’s lungs had aggregates of macrophages and lymphoplasmacytes intermixed with fewer neutrophils that expanded into the interstitium, especially around the adventitia. Immunohistochemistry with mouse antibody to recombinant H18N11 nucleoprotein revealed virus antigen in the lungs of this bat. This work represents the first study demonstrating animal susceptibility to bat influenza viruses and suggests that viral persistence up to 28 days occurs in some bats; data supporting the hypothesis that Jamaican fruit bats may be a natural reservoir host of the HL18NL11 virus. They are currently conducting additional infection studies of Jamaican fruit bats with HL18NL11 to further characterize the virology and immunology.

**Scott King** (School of Pharmacy, Department of Pharmaceutical Sciences, Regis University, Denver, CO, USA) presented the intriguing finding of antibody responses to influenza in adults with Down syndrome. Adults with Down syndrome (DS) are particularly susceptible to respiratory infections, and respiratory disease is a primary cause of death in individuals with DS over the age of 20 years. Although no studies to date have identified the pathogens responsible for the majority of respiratory infections observed in adults with DS, data from previous influenza epidemics suggest that individuals with DS have significantly higher rates of morbidity and mortality as a result of influenza infection. Data evaluating the effectiveness of immunizations in individuals with DS is limited and inconclusive, although it has been established that those with DS have decreased B cell counts. Herein, the King lab hypothesized that adults with DS will have an attenuated antibody response to immunization. Antibody titers to influenza A, influenza B and Bordetella pertussis were evaluated using a standard enzyme-linked immunosorbent assay (ELISA). Their results demonstrated that adults with DS had decreased antibody titers to influenza B and pertussis toxin. Interestingly, adults with DS had significantly increased IgG antibody titer to Influenza A compared to age matched controls (*p* value = 0.005). They hypothesize that this may be due to adults with DS acquiring a subclinical influenza A infection despite immunization.

### 2.5. Pathogen-Host Interactions

**Nicholas A. Bergren** (Colorado State University, Fort Collins, CO, USA) investigated the ability of a recombinant vesicular stomatitis-Zaire ebola virus vaccine candidate to transmit in arthropods. From 2013 thru 2016 an unprecedented outbreak of ebola virus (EBOV) occurred in West Africa. This outbreak highlighted EBOV’s ability to cause massive epidemic events and the need for a safe and efficacious vaccine that protects against ebola virus disease for health care workers and people of endemic regions. The V920 vaccine, being developed by Merck & Co., Inc., Kenilworth, NJ, USA and NewLink Genetics (Ames, IA, USA), is a live, replication-competent vesicular stomatitis virus-Indiana (VSV-I) where the VSV glycoprotein (G) gene is replaced with the glycoprotein of Zaire-EBOV (ZEBOV). The vaccine has been tested in numerous Phase 1–3 clinical trials, and the data suggest that the vaccine is generally well tolerated and effective at preventing ebola virus disease. Since the V920 vaccine candidate is constructed using the backbone of an arbovirus, studies evaluating the potential for V920 to replicate and transmit in arthropods are necessary. To accomplish this, they assessed the ability of V920 to replicate in several sandfly and mosquito cell lines, intra-thoracically (IT) inoculated and blood-fed *Aedes aegypti* and *Culex quinquefasciatus*. A wild-type VSV-I was used as a positive control and a VSV-I vectored vaccine against Semliki Forest virus (rVSV-SFV) was used as a comparator in all experiments. All viruses are replication competent in Vero cells. V920 was unable to replicate in relevant cell types or to transmit in relevant arthropod vector species, supporting a low environmental risk of this vaccine. Their work was funded by the Defense Threat Reduction Agency.

**Wendy Maury** (Dept. Microbiology and Immunology, University of Iowa, Iowa City, IA, USA) presented information concerning the control of Ebola virus infection by the polarization status of macrophages. During Ebola virus (EBOV) infection, macrophages and dendritic cells have been shown to be the first cells within the body that are virus antigen positive. Further, these cell populations are thought to support EBOV replication over the course of infection, resulting in the production of dysregulated cytokines/chemokines. As tissue macrophages are known to have a number of different phenotypes depending on their environment, The Maury lab sought to determine the effect of the phenotype on the ability of macrophages to support EBOV. Using both a BSL2 model virus of EBOV (EBOV GP/rVSV) and wild-type EBOV, they found that macrophages treated with interferon gamma (IFN-γ), causing an M1 cellular phenotype, profoundly inhibited EBOV infection. Consistent with this, IFN-γ-treated murine peritoneal macrophages transferred into a naïve mouse were protected against lethal virus challenge. In contrast, treatments that shift a macrophage towards an M2 phenotype, such as IL-4/-13, enhanced EBOV GP/rVSV infection. Enhanced infection at least in part was due to IL-4/-13-stimulated expression of C-type lectins on the surface of the cells, known surface receptors for filoviruses. Upregulation of these C-type lectins resulted in greater entry of EBOV GP/rVSV into primary peritoneal macrophages and human monocyte derived macrophages. Finally, they found that introduction of IL-4/-13-treated peritoneal macrophages into naïve mice exacerbated EBOV GP/rVSV morbidity and mortality. In total, their findings were the first demonstration that the environment of the EBOV-infected macrophage determines the permissiveness of the cell to filoviruses and supports previous findings that macrophages are a critical cell population in determining EBOV pathogenesis.

**Nathaniel Byers** (Centers for Disease Control and Prevention, Fort Collins, CO, USA) presented a method for longitudinal mosquito saliva collection for determining arbovirus vector competence that increases biosafety and throughput. The recent emergence of Zika, chikungunya and yellow-fever viruses emphasizes the need for rapid, reliable, and safe methods for analyzing vector competence from various species of mosquitoes. Arboviruses transmit from the mosquito to the vertebrate host via mosquito saliva; hence, collecting mosquito saliva to assess viral titers is an essential activity. Current collection methods are laborious and typically require sacrificing the mosquito, limiting researchers to a single saliva collection time point per insect. Inspired by the hanging blood drop and the field-oriented honey-trap method, they are designing an approach that will allow collection of saliva from individual mosquitoes during routine sugar feeds. They have systematically optimized this assay: including methods of sucrose delivery, housing, timing, and detection of virus from samples. A 3D printed lid covered with organdy turned 12-well plates into suitable habitats for both *Anopheles gambiae* and *Aedes aegypti*, as they provided a compact living space for individual mosquitoes and improved safety over cartons. To detect chikungunya virus from experimentally infected mosquitoes, quantitative reverse-transcription real-time PCR, normalized to a standard curve, was performed on RNA purified from mosquito saliva samples. This collection technique is a promising method for assaying arbovirus transmission rates over time from experimentally infected mosquitoes as it facilitates rapid longitudinal collection of infected saliva from individual mosquitoes. Also, by eliminating the need for glass capillaries and reducing manipulation of mosquitoes, this technique improves biosafety, saves time and allows for increased numbers of mosquitoes per experiment.

**Michelina Meinzer** (University of Wyoming, Laramie, WY, USA) demonstrated that climate variables and maternal antibodies can affect the natural transmission of bluetongue virus in range-pastured beef cattle. Bluetongue virus (BTV) is spread by members of the genus *Culicoides* and infects domestic and wild ruminants. It may lead to a highly fatal hemorrhagic disease in certain species, including mule deer, white-tailed deer, pronghorn, domestic sheep, and bighorn sheep. Cattle are susceptible to infection but generally asymptomatic. This study examines the effects of climate variables and maternal antibodies on the natural transmission of bluetongue virus in range-pastured beef cattle. Each summer for three seasons, 20 maternal antibody positive and 20 maternal antibody negative spring-born calves were tested twice monthly for BTV antibodies using competitive enzyme-linked immunosorbent assays and serum neutralization assays. To determine the duration of passive protection, the rate of antibody decay was determined for maternal antibody positive calves. Onset of infections was determined by seroconversion in maternal antibody negative calves and detection of BTV RNA in the blood by real time-PCR (RT-PCR), followed by virus isolation attempt from positive samples. Additionally, they collected *Culicoides* near the calves’ water source to determine weekly relative vector abundance and compared to climate variables on the day of collection as well as 2–3 weeks prior. Based on our results, maternal antibodies in calves persist to an average of 18.3 (range: 11.5–25.5) weeks of age. This likely helps protect spring-born calves from disease in high-risk years with early transmission but will play a lesser role in protecting calves when transmission occurs in late summer or fall. For the first two years of the study, BTV-17 was identified, which has historically been the predominant serotype in Wyoming.

**Alyssa Torres** (Colorado State University, Pueblo, CO, USA) presented her data on West Nile virus antibodies and dietary mercury levels in song birds collected from the Colorado Fountain Creek region. West Nile virus (WNV) is a positive strand RNA virus (*Flaviviridae*) that is transmitted by mosquitoes (*Culex* species). The virus is normally maintained and amplified in avian reservoir hosts, but infected mosquitoes will also bite other vertebrates and can result in the transmission of the virus. WNV infections have been reported all over North America, including recent infections in Colorado. Mosquitoes are routinely sampled for the presence of WNV, but bird populations are more difficult to trap and analyze. Mercury is a neurotoxin that has been shown to affect brain activity and immune response in a variety of vertebrate species, including birds. One of the most common ways for mercury to be acquired is by diet. Dietary mercury exposure has been associated with suppressed immune responsiveness in captive song birds. They trapped and banded song birds and collected blood samples in the Fountain Creek Region of Colorado in summers of 2014–2017. Blood samples are currently being screened for mercury levels using a DMA 3000 Mercury analyzer and for WNV antibodies using an indirect enzyme-linked immunosorbent assay (ELISA). They hypothesized that higher levels of mercury may correlate with WNV infection. Initial screening results show multiple birds being positive for WNV antibodies. Those same bird species have also shown higher levels of mercury than the other species. Over 800 bird blood samples have been collected over the last four years and will be analyzed for WNV antibodies and mercury. Geographic location, bird species, bird age, mercury levels, and WNV antibody titer data will be gathered and analyzed for any correlations between WNV exposure and blood mercury concentrations.

**Molly Butler** (Colorado State University, Fort Collins, CO, USA) identified a retrovirus that is associated with thymic lymphoma in Gunnisons’s prairie dogs (*Cynomys gunnisoni*). The Gunnison’s prairie dog is one of five species of prairie dogs native to North America. Prairie dogs are recognized as “keystone” species in the ecosystem of Colorado, providing habitat for numerous ground-dwelling birds and mammals, and serving as the major food source for several carnivorous species including the endangered black-footed ferret. The most significant factor affecting survival of prairie dog species in Colorado is epizootics of plague (caused by *Yersinia pestis*). Prairie dogs are highly sensitive to plague infection, with death occurring rapidly in infected animals with no apparent clinical signs prior to death from acute septicemia. Although all populations of prairie dogs are at risk for plague die-offs, Gunnison’s prairie dogs have been extensively studied in Colorado. As part of plague research and management efforts in Colorado, prairie dogs were captured from several colonies across the state. Animals found dead or that died under anesthesia were given a full necropsy to determine cause of death. As part of this effort, three Gunnison’s prairie dogs from Colorado were diagnosed with thymic lymphoma. No other neoplastic disorders were identified in any of the prairie dogs examined. We investigated the possibility that the cases of thymic lymphoma observed in Gunnison’s prairie dogs were associated with retroviral infection. RNA was isolated from the tumors and spleens from these animals and from spleens collected from two prairie dogs without a visible mass. Real time-PCR (RT-PCR) using degenerate primers targeting conserved amino acid sequences (LPQG and YMDD) in the reverse transcriptase gene was used to identify novel retroviral sequences from animals with thymic lymphoma. Retroviral sequences were not amplified from the spleens of animals without a mass. NextGen sequencing of RNA samples from tumor-positive and tumor-negative prairie dogs yielded a retrovirus-like sequence which has a complete open reading frame (ORF) encoding for *gag*, *pro-pol*, and *env*. The greatest number of reads of this sequence was found in tumor samples, in lower numbers in spleen samples from tumor-positive prairie dogs, and was not found in tumor-negative prairie dog spleen samples. This represents a candidate for a novel retrovirus associated with thymic lymphomas in prairie dogs.

**Elliott Chiu** (Colorado State University, Fort Collins, CO, USA) presented a study that demonstrated Feline leukemia virus replicates faster in mountain lion cells. Feline leukemia virus (FeLV) is a common domestic cat disease that has been documented in a range of wild felids. While the progression of FeLV infection has been well documented in the domestic cats, infection has not been thoroughly investigated in atypical hosts. In domestic cats, four types of infection (abortive, latent, regressive, and progressive) have been documented. It has been hypothesized that the presence and activity of the endogenous form of the virus (enFeLV) account for some differences in disease phenotypes. Since the early 2000’s, Florida panthers (*Puma concolor coryi*) have experienced two FeLV outbreaks, resulting in at least 29 unique infections. As Florida panthers lack enFeLV and that viruses in naïve hosts may often be more virulent that in native hosts, they hypothesized that FeLV viral load, proviral load, and viral replication will be greater in panthers compared to domestic cats. They measured viral and proviral tissue loads by quantitative PCR (qPCR) in domestic cats and panthers following natural and experimental infections in vivo as well as experimental infections in vitro. Fibroblasts derived from panthers showed accelerated FeLV viral replication rates compared to domestic cat fibroblasts, resulting in higher proviral load and productive viremia. Endpoint naturally occurring FeLV-A infection in panthers had similar tissue viral loads to experimentally infected cats in a small set of animals. Our research documents that FeLV-A can replicate with accelerated kinetics in panther cells in vitro, but endpoint viral loads of panthers may be similar to that observed in domestic cats.

**Emily Feldman** (University of Colorado, Boulder, CO, USA) described a project designed to harness the genetic diversity of captive owl monkeys to study the special HIV variants that seed new human infections. Human immunodeficiency virus-1 (HIV-1) transmission to a new individual elicits a strong selective bottleneck on the existing virus population, and typically only one or few very special viral variants establish each new infection. Only very recently have these transmitted/founder (T/F) HIV-1 variants been appreciated for their unique ability to initiate an infection. The vast majority of HIV-1 research has been performed utilizing lab-adapted or late-stage HIV-1 variants, which do not recapitulate the cell-tropism, unique resistance to interferon and restriction factors, and antigenic properties of T/F HIV-1 variants. Critically, the current animal model for HIV-1 infection and vaccine research, the rhesus macaque, does not support the replication of T/F HIV-1. Many of the T/F HIV-1 variants are highly restricted by the macaque version of cluster of differentiation 4 (CD4), the HIV-1 entry receptor. Additionally, macaques block T/F HIV-1 replication at stages post-entry due to several restriction factors with activity against HIV-1. Excitingly, their lab discovered the first monkey, the Spix’s owl monkey, which encodes a CD4 that is broadly permissive for major global subtypes of T/F HIV-1. Further, owl monkeys are only known to encode a minimal number of restriction factors that block HIV-1, one of which (TRIM-Cyp) is evaded with a single point mutation in the HIV-1 genome, while another (tetherin) is polymorphic in owl monkeys with some individuals encoding ineffective alleles. They harnessed the genetic diversity of multiple owl monkey colonies to define key molecular interactions of T/F HIV-1 biology. Their preliminary data demonstrated that these studies will expand our understanding of T/F HIV-1 biology.

**Elena Judd** (University of Colorado, Boulder, CO, USA) presented a study that investigated the evolution of the interferon response across primates. Interferons and interferon-stimulated genes are involved in a variety of mechanisms that suppress viral replication within host cells. Upon recognition of a virus, a signaling cascade leads to the expression of secreted interferons. Interferons are then taken up by neighboring cells, where they signal the increased expression of hundreds of downstream genes that produce an antiviral cellular state. In turn, viruses have evolved strategies to subvert the interferon response by evading recognition, impairing signaling, or hindering restrictive functions. Induction genes and interferon-stimulated genes must evolve in turn to maintain an effective defense against viruses in spite of high viral mutation rates. The tit-for-tat evolution between viruses and host proteins creates a situation of coevolution. In this study, the Sawyer lab scanned interferon induction genes and interferon-stimulated genes for signatures of natural selection using alignments of primate gene sequences and the Phylogenetic Analysis by Maximum Likelihood (PAML) program. This program uses different models of nucleotide substitution to detect loci which have accumulated significantly elevated rates of nonsynonymous (amino acid altering) substitutions. They analyzed one hundred interferon-stimulated genes and sixty interferon induction genes for positive selection. Interestingly, interferon-stimulated genes had a significantly elevated rate of nonsynonymous mutations when compared to the genes involved in interferon induction, and when compared to a set of random genes. Induction genes were not elevated significantly when compared to the null set. Rapid evolution may indicate that viruses are enhancing selective pressure for new mutations, specifically in interferon-stimulated genes, and could provide a greater understanding of the host proteins important for restriction of viral infection.

**Laura Hoon-Hanks** (Colorado State University, Fort Collins, CO, USA) presented their findings regarding a respiratory disease in pythons experimentally infected with python nidovirus and other updates on this emerging veterinary disease. A severe respiratory illness of pythons has been observed by veterinarians since the 1990s. Circumstantial evidence has linked a novel python nidovirus to the disease. They conducted an experimental infection in ball pythons (*Python regius*) to test the hypothesis that ball python nidovirus (BPNV) infection would yield clinical signs and histologic lesions consistent with respiratory disease. Additionally, they have investigated natural disease in mixed snake colonies, providing further perspectives on this emerging veterinary disease. Five juvenile ball pythons were inoculated: three with BPNV-infected medium and two with uninfected medium. Antemortem swabs were performed weekly and tested for BPNV RNA by PCR. Euthanasia and postmortem examination were performed on infected snakes at 5 weeks, 10 weeks, and 12 weeks post-inoculation (PI) based on clinical signs. The most significant lesions in the infected snakes included chronic-active catarrhal rhinitis, stomatitis, tracheitis, and esophagitis with variable epithelial proliferation and an interstitial and proliferative pneumonia. Infectious virus was recovered from swabs and tissues from infected snakes. Control snakes remained negative throughout the experiment and did not show clinical signs or share histologic lesions. Their findings establish a causal relationship between BPNV infection and respiratory disease in ball pythons. BPNV is part of an expanding group of related viruses that have been associated with respiratory disease in reptiles and mammals. This work, as well as their additional studies, has revealed insights into clinical course, possible routes of transmission, useful diagnostics, viral genomic diversity, species specificity, and disease epidemiology of the python nidovirus.

**Maryska Kaczmarek** (University of Colorado, Boulder, CO, USA) demonstrated using their results that C-C chemokine receptor type 5 (CCR5) is under recurrent positive selection, making some primates resistant to infection by immunodeficiency viruses (human immunodeficiency virus (HIV) and simian immunodeficiency cirus (SIV)). Cluster of differentiation 4 (CD4) and CCR5 are the receptor and co-receptor that allow HIV and SIV to enter cells. CD4 has been shown to be under recurrent positive selection, which has contributed to differences in primate susceptibility to immunodeficiency virus entry. The evolution and functional relevance of species-specific differences in CCR5 has largely been overlooked, partly due to the primary focus on CD4 as the cellular receptor responsible for differences in viral entry. Here, they performed an evolutionary analysis with a large dataset of primates and show that primate CCR5 also bears the signature of recurrent positive selection, particularly within the New World Monkey clade. In addition, they have run site-specific models of positive selection that identified sites in the N-terminus of CCR5, which are highly variable within New World Monkeys. Amino acids in this region are sulfated and this modification is known to be required for viral entry. Interestingly, they show that the sulfation motif is absent in New World monkeys, and this contributes to lower levels of entry when a permissive CD4 is paired with a CCR5 from New World monkeys. New World Monkeys are not known to harbor any modern viruses related to HIV and SIV, this suggests the compelling hypothesis that New World monkeys have acquired adaptive mutations in CCR5 that protect them from these viruses. Together, their work shows evidence that CCR5 can pose as a barrier to cross-species transmission in primates.

**Rebekah McMinn** (Colorado State University, Fort Collins, CO, USA) expanded on the knowledge base of Middle East respiratory syndrome coronavirus cross-species transmission through studying the viral spike plasticity in the context of the common vampire bat (*Desmodus rotundus*) dipeptidyl peptidase 4 (DPP4) receptor. In 2012, a novel coronavirus, Middle East respiratory syndrome coronavirus (MERS-CoV), was discovered in humans and dromedary camels, although genetic evidence supports a bat ancestor. This range of animal hosts lead them to hypothesize that MERS-CoV can readily adapt to new hosts. The receptor for MERS-CoV, DPP4, has previously been shown to act as a species barrier. By passing the virus over time on cells stably expressing the common vampire bat DPP4 receptor, which MERS-CoV binds to inefficiently, they aimed to determine how potential adaptation in the spike glycoprotein may influence species tropism. They showed that observed adaptations that arise in the viral spike protein at residues 465 and 510 after extended replication in cells expressing the bat DPP4 receptor. Protein structures that model electrostatic potential across the surface were used to facilitate preliminary analysis of the adapted MERS-CoV Spike variants. Viral genomes containing the relevant mutations are being created through a reverse genetics system for further testing on binding affinity and growth potential. The ability of the MERS-CoV spike to adapt to diverse host species receptors may play a significant role in cross-species transmission.

**Megan Miller** (Colorado State University, Fort Collins, CO, USA) used experimental infections to determine if a sexual transmission model for Zika virus is possible. Zika virus (ZIKV) is a mosquito borne flavivirus that has been prevalent in Asia, parts of Africa, and Central and South America. The recent pandemic in South and North America resulted in over 1,000,000 cases. The most common symptoms of Zika virus disease are rash, arthralgia, conjunctivitis and headache; however compilations have been observed, including Guillain-Barre syndrome and birth defects, in infected newborns. Interestingly, ZIKV is the only known flavivirus to be able to transmit sexually. This study was undertaken to determine an immunecompetent, non-primate, small animal model that would be valuable for understanding the mechanism of ZIKV sexual transmission. Previous published evidence showed that cottontail rabbits seroconvert after inoculation with ZIKV, and other infection studies suggested that both guinea pigs and the Jamaican fruit bats develop viremias and seroconvert after inoculation. They are looking for evidence of infections in male tissues and body fluid of these three animal models; rabbits (NZW strain), guinea pigs (Hartley strain) and the local colony of *Artibeus jamaicensis* (Jamaican fruit bat). Groups of each species are being inoculated with ZIKV or uninfected cell culture media, and then blood, oral swab, urine and semen samples are collected and tested for ZIKV by quantitative real time-PCR (qRT-PCR) and plaque assays. To date, they have determined that NZW rabbits are not a competent model for ZIKV sexual transmission. Additional results are pending. By determining an immune-competent, non-primate, sexual transmission animal model for ZIKV they hope to begin addressing many unknown questions about this mode of transmission and its influence on ZIKV disease.

**Jessica Costlow** (Colorado State University, Pueblo, CO, USA) presented their exploration of cancer metabolic inhibitor drugs as potential anti-viral treatments for alphaviruses. Alphaviruses infect millions of people and animals. Chikungunya virus (CHIKV) is an arthritogenic alphavirus that is currently outbreaking. Sindbis Virus (SINV) is a relative to CHIKV and acts as a widely accepted model to study alphavirus infections. Viruses are obligate intracellular parasites and dependent on the host cell. Alphaviruses take over the host cell and make dramatic changes to the normal cell physiology in a number of cellular pathways, including an upregulation in glycolysis. Cancerous cells also display an elevated metabolic phenotype which directly correlates to cancer development and metastasis. There are several US Food and Drug Administration (FDA)-approved cancer drugs that target this elevated metabolic rate and reduce it to normal levels and thereby slow or prevent cancer growth. Two common cancer drugs listed by the National Cancer Institute that inhibit metabolism are Imatinib and Dichloroacetate (DCA). They hypothesized that these FDA-approved cancer metabolic inhibitor compounds could be repurposed and used to treat virally infected cells. Since some Alphaviruses have been shown to require an elevated metabolic rate for optimal replication, these drugs may reduce metabolism and prevent viruses from replicating as effectively. Their results indicated that treatment of SINV-infected Baby Hamster Kidney (BHK) cells with Imatinib and/or DCA show reduced viral growth kinetics. Treatment with 12 μM of imatinib reduced the percentage of infected cells by approximately 45% at 24 h post treatment, while maintaining normal cell viability on uninfected cells. DCA treatment reduced viral titers but had to be used at much higher concentrations (greater than 50 μM) to have any effect on infected cells as compared to imatinib treatment. Further testing is currently ongoing to determine the optimal concentrations for DCA and imatinib to reduce viral replication and still maintain cell viability. These results are promising and we are currently investigating more details on the utility of these drugs as an anti-viral therapy.

**Carmen Ledesma-Feliciano** (Colorado State University, Fort Collins, CO, USA) presented work on a vaccine system and found that Feline foamy virus Bet can be replaced by feline immunodeficiency virus Vif in a novel chimeric vaccine system. Feline foamy virus (FFV) is a Spumavirus that has not been associated with a disease syndrome in domestic cats despite life-long infection. Because of this, FFV has potential use as a vaccine and gene therapy vector. In order to test the ability of a replicative novel vaccine candidate to infect and induce an immune response, we inoculated SPF domestic cats (*n* = 4/group) with wild-type (wt) FFV or a chimeric FFV-Vif virus containing the feline immunodeficiency virus (FIV) *vif* gene replacing the FFV *bet* gene. Vif and Bet proteins counteract intrinsic feline APOBEC3 (feA3) restriction factor proteins through different mechanisms. FFV infection was confirmed in wt-inoculated cats as early as 21 days post-infection via PCR of peripheral blood mononuclear cell (PBMC) DNA. Chimera-inoculated cats yielded either negative or indeterminate results by day 53 post-inoculation. At this point, chimera-inoculated cats were re-inoculated: two received more chimeric virus and two wt FFV. One of the cats re-inoculated with wt virus was consistently FFV-positive 24 days following re-inoculation (PCR). Antigen-specific ELISA demonstrated that all wt and chimera-inoculated cats seroconverted against FFV-Gag by day 28 post-inoculation. Quantification of anti-Gag immune response over time showed an increasing titer in wt-inoculated cats that either continued to increase or plateau. Chimera-inoculated cats had comparable anti-Gag antibody titers following re-inoculation. The four cats initially inoculated with wt FFV seroconverted against Bet by 42 days post-inoculation, while the two chimera-inoculated cats later exposed to wt FFV seroconverted by 45 days post-re-inoculation. Anti-Vif seroconversion in three chimera-inoculated cats was detected 15 days post-inoculation and increased following re-inoculation with either wt or FFV-Vif. These findings demonstrate that this chimeric vector system can induce a detectable immune response in domestic cats comparable to wt infection, and that FFV is capable of superinfection.

**Joseph Lopez** (Colorado State University, Pueblo, CO, USA) continued the theme of viral targets and therapies by presenting their work on the impact of Cannabinoid Receptor (CB1) Antagonism/Agonism on Alphavirus Replication. Cannabinoid receptors are found on many cells throughout the body. Cannabinoids bind to the receptors and initiate a signaling cascade within the cell. It has been shown that activation of cannabinoid receptors (CB1) alter cellular physiology and increase anabolic pathways such as fatty acid and glucose synthesis. Viruses are obligate intracellular parasites that require a host cell to provide essential macromolecules, nutrients, and energy for replication. Alterations to metabolic pathways directly impact the ability of the virus to successfully replicate and produce new virions. They hypothesized that activation or inhibition of the CB1 receptor/endocannabinoid signaling pathway will cause specific changes in cellular physiology which will directly impact alphavirus infection in cultured cells. To investigate their hypothesis, they utilized murine and human cell lines to measure the impact of the endocannabinoid system on alphavirus infection. At this point, all testing has been performed with the prototype alphavirus, which is Sindbis virus. This is a plus strand RNA virus that is transmitted by infected mosquitoes. SINV is a safe virus to be used for testing in a lab setting, but it is closely related to other mosquito viruses that are causing outbreaks around the world, including Chikungunya virus. All cells being used have been confirmed to have CB1 receptors on the cells. They have tested the activation of CB1 receptors (using the agonist/activator ACEA) and the inhibition of the receptors (using the antagonist/inhibitor AM251) and have found that treatment with either the agonist or antagonist cause significant changes to virus replication at 24 h post infection. They are also investigating the change in CB1 receptor expression during chronic viral infection to measure different susceptibilities that may be influenced by the endocannabinoid system. Specific details and results will be discussed.

**Amy MacNeill** (Colorado State University, Fort Collins, CO, USA) gave a presentation on their work about the Safety of Recombinant Myxoma Virus Therapy in Dogs with Soft Tissue Sarcomas. Many oncolytic viruses that are efficacious in murine models of cancer are ineffective in humans. The outcomes of oncolytic virus treatment of dogs with spontaneous tumors may be better predictors of human cancer response to treatment and may improve treatment options for dogs with cancer. The objective of this study was to evaluate the safety of intratumoral injection of myxoma virus lacking the serp2 gene (MYXVΔserp2) in dogs with spontaneous subcutaneous soft tissue sarcomas. To achieve this, MYXVΔserp2 was injected intratumorally in five dogs with spontaneous soft tissue sarcoma. Tumor volume, tumor biopsies, and blood, urine, feces, saliva, and swabs from the injection site of the virus were collected at several time points following the injection of MYXVΔserp2 to evaluate organ function, immune response, and virus distribution. Additionally, post-operative MYXVΔserp2 was administered to four dogs with grade II-III soft tissue sarcoma to determine if virotherapy would decrease tumor recurrence. To date, no adverse effects have been observed in any canine cancer patients following the MYXVΔserp2 therapy. No clinically significant changes in complete blood profiles, serum chemistry analyses, or urinalyses were measured. Virus was detected by PCR in some biopsied tumors, but virus shedding was not observed. Anti-MYXV antibodies were rarely detected in dogs. These findings provided needed safety information to advance clinical trials using MYXVΔserp2 to treat dogs with cancer. Future studies were outlined that would help determine if oncolytic virotherapeutics that are efficacious in dogs are effective in human cancer patients.

**Candace K. Mathiason** (Department of Microbiology, Immunology and Pathology, Colorado State University, Fort Collins, CO, USA) explored the natural transience of reproductive microbiota and placental immune cell profiles on transplacental trafficking of infectious agents. Maternal infections during pregnancy induce immune factors and present infectious agents to the maternal-fetal interface. Prenatal exposures to infectious pathogens are increasingly recognized to play an important etiologic role in the development of fetal disorders with lifelong effects, i.e., zika—severe brain defects including microcephaly; influenza—marginalized immune responses, depression, and schizophrenia; prions—asymptomatic carrier state. The hallmark of successful pregnancy includes a maternal immunological profile skewed towards an anti-inflammatory response. Disruption of microbiota has been linked to proinflammatory responses. It is known that the decidua immune cell profile and the placental microbiome community transition throughout pregnancy. Yet, the relationships between these responses and the impact their interactions have on transplacental trafficking are unknown. To investigate the role altered placental microbiome and immune responses may play in facilitating or inhibiting the transfer of infectious agents from mother to baby The Mathiason lab employ direct use of: (i) reproductive tissue (uterus, placenta, vagina) microbiome analysis; (ii) decidua/fetal membrane flow cytometric specific immune cell profile analysis; (iii) placental organotypic slice cultures and (iv) 3-D culture of primary trophoblast cells. The overarching goal of this work was to answer the question: How does the natural transience of the placental microbiome and/or immune cell profile exacerbate or hinder pathogen transfer from mother to baby, and how can we take advantage of this to better design preventative/therapeutic strategies?

### 2.6. Mentoring

**Erica Suchman** (Colorado State University, Fort Collins, CO, USA) presented her methods of teacting virology that she has developed from nearly two decades on-site experience, and also from her interactions with the Committee on Technology Enhanced Education for the American Society of Microbiology (Chairperson), the MicrobeLibrary, the American Society of Microbiology’s web based peer reviewed education resource collection (Editor-in-chief, 2003–2012), and membership of both the ASM Education Board and ASM committee on International Education. In her talk, mechanisms for increasing student engagement and learning in both a large lecture and smaller laboratory virology course were presented and discussed. In her current lectures, students analyze trends in taxonomy for RNA and DNA viruses, concept mapping, and group exams, to move past memorization. In the laboratory course students use pre-lab quizzing to prepare for daily labs, make predictions based on phylogenetic trees, and perform blast searches on sequences to determine the identity of assigned unknown viruses that augment laboratory activities. Examples and materials used in each type of teaching environment were presented.

## References

[1] Gilden D., White T., Khmeleva N., Heintzman A., Choe A., Boyer P.J., Grose C., Carpenter J.E., Rempel A., Bos N. (2015). Prevalence and distribution of VZV in temporal arteries of patients with giant cell arteritis. Neurology.

[2] Nagel M.A., White T., Khmeleva N., Rempel A., Boyer P.J., Bennett J.L., Haller A., Lear-Kaul K., Kandasmy B., Amato M. (2015). Analysis of varicella-zoster virus in temporal arteries biopsy positive and negative for giant cell arteritis. JAMA Neurol..

[3] Gilden D., White T., Khmeleva N., Boyer P.J., Nagel M.A. (2016). VZV in biopsy-positive and -negative giant cell arteritis: Analysis of 100+ temporal arteries. Neurol. Neuroimmunol. Neuroinflamm..

[4] Jones D., Alvarez E., Selva S., Gilden D., Nagel M.A. (2016). Proinflammatory cytokines and matrix metalloproteinases in CSF of patients with VZV vasculopathy. Neurol. Neuroimmunol. Neuroinflamm..

[5] Jones D., Neff C.P., Palmer B.E., Stenmark K., Nagel M.A. (2017). Varicella zoster virus-infected cerebrovascular cells produce a proinflammatory environment. Neurol. Neuroimmunol. Neuroinflamm..

[6] Campagnola G., McDonald S., Beaucourt S., Vignuzzi M., Peersen O.B. (2015). Structure-function relationships underlying the replication fidelity of viral RNA-dependent RNA polymerases. J. Virol..

